# Managing pain in HIV/AIDS: a therapeutic relationship is as effective as an exercise and education intervention for rural amaXhosa women in South Africa

**DOI:** 10.1186/s12889-021-10309-7

**Published:** 2021-02-05

**Authors:** Kirsty Jackson, Antonia L. Wadley, Romy Parker

**Affiliations:** 1 Division of Physiotherapy, Department of Health and Rehabilitation Sciences, University of Cape Town, Observatory, Cape Town, 7925 South Africa; 2 Zithulele Hospital, Eastern Cape Department of Health, Mqanduli, South Africa; 3 Brain Function Research Group, School of Physiology, Faculty of Health Sciences, University of the Witwatersrand, Johannesburg, South Africa; 4 Pain Management Unit; Department of Anaesthesia and Perioperative Medicine, Neuroscience Institute, University of Cape Town and Groote Schuur Hospital, Observatory, Cape Town, 7925 South Africa

**Keywords:** HIV/AIDS, Pain, Therapeutic relationship, Exercise, Education

## Abstract

**Background:**

Pain is one of the most prevalent symptoms in people living with HIV/AIDS and is largely undermanaged. Both a peer-led exercise and education Positive Living programme (PL programme) and the PL programme workbook alone were previously found to be effective in reducing pain in urban amaXhosa Women Living With HIV/AIDS (WLWHA). A therapeutic relationship was hypothesised to have contributed to the efficacy of both interventions. The aim of the study was to determine the effectiveness of the PL programme and a therapeutic relationship, compared to a therapeutic relationship alone in managing pain amongst rural amaXhosa WLWHA on pain severity and pain interference, and secondary outcomes, symptoms of depression, health-related quality of life (HRQoL) and self-efficacy.

**Methods:**

In this two-group, single-blind, pragmatic clinical trial with stratified convenience sampling, the PL programme and therapeutic relationship, was compared to a therapeutic relationship alone in rural amaXhosa WLWHA. The PL programme was a 6-week, peer-led intervention comprising education on living well with HIV, exercise and goal setting. The therapeutic relationship comprised follow-up appointments with a caring research assistant. Outcome measures included pain severity and interference (Brief Pain Inventory), depressive symptoms (Beck Depression Inventory), HRQoL (EuroQol 5-Dimensional outcome questionnaire) and self-efficacy (Self-efficacy for Managing Chronic Disease 6-Item Scale). Follow-up was conducted at 4, 8, 12, 24, and 48 weeks. Mixed model regression was used to test the effects of group, time, and group and time interactions of the interventions on outcome measures.

**Results:**

Forty-nine rural amaXhosa WLWHA participated in the study: PL group *n* = 26; TR group *n* = 23. Both intervention groups were similarly effective in significantly reducing pain severity and interference and depressive symptoms, and increasing self-efficacy and HRQoL over the 48 weeks. A clinically important reduction in pain severity of 3.31 points occurred for the sample over the 48 weeks of the study. All of these clinical improvements were obtained despite low and suboptimal attendance for both interventions.

**Conclusions:**

Providing a therapeutic relationship alone is sufficient for effective pain management amongst rural amaXhosa WLWHA. These findings support greater emphasis on demonstrating care and developing skills to enhance the therapeutic relationship in healthcare professionals working with rural amaXhosa WLWHA.

**Trial registration:**

PACTR; PACTR201410000902600, 30th October 2014; https://pactr.samrc.ac.za.

**Supplementary Information:**

The online version contains supplementary material available at 10.1186/s12889-021-10309-7.

## Background

Pain is reported by 54–83% of people living with HIV/AIDS (PLWHA), and is typically of moderate to severe intensity [for review, see Parker et al. 2014] [1]. HIV-associated pain varies in type and cause. HIV-associated neuropathic pain may occur as a direct or indirect consequence of the virus, or be secondary to treatments for the virus. HIV-associated nociceptive pain may arise due to acute tissue damage, injury or complications occurring secondary to immune failure. HIV-associated chronic nociplastic pain may arise due to the direct or indirect effects of the virus on the nervous system [1–3]. Pain in PLWHA, similar to pain in other chronic conditions, is influenced by a range of biomedical factors such as co-morbidities and immune dysregulation, and a range of psychosocial factors such as poverty, level of education, mood disorders, social isolation and the double burden of living with both HIV and chronic pain, which are both stigmatized conditions [3].

Pain in PLWHA is under-treated, partly because no feasible effective pharmacological intervention and guidance is currently available [1, 4]. The limited efficacy of pharmacological treatments for pain in this population necessitates developing specific pain management interventions to manage pain in PLWHA and investigation of non-pharmacological approaches is warranted [1, 5, 6]. Non-pharmacological interventions that have been investigated for managing pain in PLWHA include education, self-management interventions, cognitive behavioural therapy (CBT), physical exercise, and a peer-led exercise and education intervention, the Positive Living (PL) programme, which incorporates the before-mentioned therapies in an effective multimodal treatment strategy [7–12]. The evidence supports the use of multimodal approaches for managing pain [7–12].

The PL programme, a six-week, peer-led exercise and education intervention, incorporating the principles of CBT, was developed as a treatment option for managing pain in HIV [8]. The programme was found to be more effective for managing pain than standard care in a study of urban amaXhosa WLWHA in a resource poor community in Cape Town, South Africa [7, 8]. Prior to participating in this previous intervention study, the women had received standard care over 15 months and had shown no changes in pain intensity or interference [7]. Over the 4 months of the study, the results of Parker et al. showed that pain severity and pain interference with function were significantly reduced in all the women who participated in the study (the control group, who continued with usual care and were provided with the workbook for the PL programme, and those who participated in the PL programme) [7]. Many studies support that the therapeutic relationship which develops between patients and health care professionals or between participants and researchers over the course of a study, has an effect in optimising health outcomes in patients [13, 14]. Acknowledging the effect that a therapeutic relationship, which was possibly established over the course of follow-up visits, may have had on health outcomes [15], Parker et al. [7] suggested that the treatment effect of therapeutic relationships be considered in future studies on interventions for managing pain in WLWHA.

Given the efficacy of the PL program in urban amaXhosa WLWHA for South Africa, we wished to investigate its efficacy in rural amaXhosa WLWHA. Additionally, we wished to determine whether the therapeutic relationship alone (without the addition of the educational workbook of the PL programme) was as effective at improving pain outcomes as the PL programme. We conducted a single-blind pragmatic clinical trial to determine the efficacy of the PL programme and a therapeutic relationship (PL intervention), compared to a therapeutic relationship alone (TR intervention) in rural amaXhosa WLWHA. The primary outcomes were pain severity and pain interference, and secondary outcomes were symptoms of depression, HRQoL and self-efficacy.

## Methods

A two-group, single-blind pragmatic clinical trial, with stratified convenience sampling, was conducted, to compare the PL programme and a therapeutic relationship (PL intervention) to a therapeutic relationship alone (TR intervention). The research assistant (RA) who conducted data collection was blinded to participants’ group allocation. Follow-up was conducted at Weeks 4, 8, 12, 24 and 48, in keeping with the pilot study [7], to record change over time and to establish if any effects were maintained over a longer period of time.

Ethical approval was obtained from the Faculty of Health Sciences, Human Research Ethics Committee, University of Cape Town (HREC REF: 932/201) and the Eastern Cape Department of Health (Ref: EC_2015RP30_713). A sample of convenience of amaXhosa WLWHA was recruited from two HIV outreach clinics served by Zithulele Hospital in the Eastern Cape, South Africa, a deeply rural, resource-poor environment. The Pumalanga Clinic and Ngcwanguba Community Health Centre (CHC), were chosen as sites for recruitment, intervention and data collection as each is well situated with frequent public transport services. This facilitated patient access by minimising barriers to care and loss to follow up, in order to provide an understanding of the impact of the interventions without confounding from other barriers to access. A stratified, convenience sampling model was used to ensure equal representation of women attending the two clinics resulting in two strata. As this was a pragmatic study, exploring the efficacy of an intervention in a specific population in a rural area where clinical attendance is challenging, convenience sampling was then used to select women from each of the strata who were willing to participate in the study. The sample was not intended to represent all amaXhosa women LWHA in rural areas of South Africa. Any women who met the following inclusion criteria were invited to participate in the study: ambulant, HIV-positive, amaXhosa and aged 18–40 years, attending Pumalanga Clinic or Ngcwanguba CHC for anti-retroviral therapy (ART) or being monitored for their condition at the clinic. They also needed to answer “yes” to the first question on the Brief Pain Inventory (BPI) [16]: “Throughout our lives most of us have had pain from time to time (such as minor headaches, sprains, toothaches). Have you had pain other than these everyday kinds of pain during the last three months?”. Exclusion criteria were: on ART but had not yet been on the present treatment regimen consistently for three months, previously participated in an education and/or exercise intervention to instil skills for managing pain, considered unfit for exercise according to the American College of Sports Medicine (ACSM) guidelines [17, 18], found to have a cognitive impairment or moderate to severe intellectual disability as assessed by a medical officer or occupational therapist using clinical judgement and the Mini Mental State Examination or Allen Cognitive Level Screen Assessment where appropriate.

Sample size was calculated using a conservative minimum detectable difference in pain severity, of a change in three points on the Visual Analogue Scale (VAS) (0–10) on the BPI [19, 20]. To detect such a difference, and using a conservative standard deviation of three, each intervention group required a sample of 13 to provide a power of 91% and yield statistically significant results (*p* < 0.05). A larger sample of 24 participants for each intervention group was chosen to allow for attrition in each group and for two appropriately sized PL programme groups of 12 participants in each to be set up, based on the prior study [7, 8]. Consequently, we aimed for a sample size of 48, comprising 24 participants in each intervention group.

All participants received the therapeutic relationship intervention. In addition, the PL intervention group received the PL programme. The RA remained blind to the participants’ allocated intervention group throughout the study and confirmed this post study.

### Therapeutic relationship intervention

Due to the potential benefits of the relationship that forms between participant and RA resulting from repeated interaction during data collection [7], it was acknowledged that a control group would be providing a therapeutic relationship intervention and measuring the effect of this. The measurement of outcome measures by a RA was, therefore, reclassified as the therapeutic relationship intervention as we purposefully endeavoured to measure the potential benefits of this interaction.

A therapeutic relationship was purposefully developed by the RA between herself and participants by introducing and maintaining a caring relationship as a continuous intervention over the study period. These interactions included meeting with participants at each data collection point to complete interview-administered questionnaires, which took place at Pumalanga Clinic and Ngcwanguba CHC, as well as telephonic communication to encourage data collection attendance, enquiring after participants in the case of data collection absenteeism and any follow-up done telephonically.

The RA was identified by researchers from a local non-profit organisation. She was amaXhosa, had previous experience as a RA and displayed innate ability to be empathetic, which training was focused on further enhancing. The primary investigator (KJ) facilitated the development of interpersonal skills, communication skills and self-awareness for the RA to be able to purposefully show care and generate and maintain empathy and a therapeutic relationship over the 48 weeks of the study. The RA further developed the ability to demonstrate care by maintaining culturally appropriate respect during all interactions with participants, listening for verbal cues and being aware of non-verbal cues, reflecting on participants’ communication with her and responding back to participants appropriately [21]. To facilitate the ability to maintain the therapeutic relationship over the study, the RA participated in a self-reflection and debriefing process with the primary investigator after each data collection day. Additionally, support was available from a social worker if necessary.

### PL programme

Participants in the peer-led PL programme, guided by the PL workbook (provided in both English and isiXhosa), met over six consecutive weeks for two hours per week in a venue nearby the Pumalanga Clinic. The sessions covered six topics: self-management and exercise, managing common symptoms of HIV/AIDS, stress management, pain, eating well, and continuing as a self-manager. A facilitated group discussion on each educational topic, development of action plans, exercise routine (from Week two) and practice of a relaxation strategy were incorporated into each session. To commence Week one of the programme, the group discussed confidentiality and signed mutually agreed contracts to show commitment to attending the group. Following weekly facilitated discussions on each educational topic, participants would develop a related action plan. Action plans were reviewed at the next session, and a weekly review of the exercise action plan was facilitated over the programme. The 20-min exercise routine provided in the workbook was followed, and increased weekly by an additional two minutes of an exercise chosen by group members. Developing, adhering to, and testing action plans, increasing time spent on physical exercise, and learning the art of performing relaxation, with peer support, was aimed at facilitating skill development, to improve self-efficacy [22, 23]. Regular follow-up with participants took place to remind them of scheduled group sessions and with those who had been absent to further encourage attendance.

Prior to commencing the PL programme, peer-leader training by the primary investigator took place over 40 h during January and February 2015 at Zithulele Hospital and Pumalanga clinic. Training was facilitated by training assistants, as the primary investigator was not fluent in isiXhosa. One of the roles of the training assistants was to aid communication and understanding between the primary investigator and the peer-leader by interpreting when necessary. The other was to ensure that a high standard of facilitation was met and that the content of the workbook was well maintained during PL programme facilitation by the peer-leader. Prospective peer-leaders were identified by the managers of the Zithulele Hospital ART programme. The chosen peer-leader for facilitating the PL programme was an amaXhosa woman, HIV-positive, well-respected by community members and experienced in peer-education for PLWHA.

### Instrumentation

Validated isiXhosa versions of all outcome measures were used. The primary outcomes, pain severity and pain interference, were measured on the BPI-Xhosa, which generates a pain severity score (PSS) and pain interference score (PIS) [16]. The BPI was designed for research and endorsed by the Initiative on Methods, Measurement, and Pain Assessment in Clinical Trials (IMMPACT) guidelines for pain studies [19, 24]. It has been commonly used to assess pain in PLWHA [1]. As per the English version, the BPI-Xhosa is an appropriate outcome measure of pain severity and pain interference and was found valid and reliable, with good internal consistency, for amaXhosa WLWHA in urban areas in South Africa and used to study change in pain over time in such populations [25].

The PSS is the average of scores on pain at its “worst”, “least”, “average” and “right now”, reflecting the variable nature of pain severity. The PIS is determined by an average of scores reflecting the interference of pain on seven domains of function including walking, general activity, work, relationship with others, enjoyment of life, mood and sleep. Questions are scored by numeric rating scale (NRS) from 0 to 10, where for pain severity ‘0’ represents ‘no pain’ and ‘10’ represents the ‘pain as bad as you can imagine’. For pain interference ‘0’ on the NRS represents ‘does not interfere’ and ‘10’ represents ‘completely interferes’ [16].

It is recommended, by the IMMPACT group, that studies evaluating the efficacy of interventions on pain, should use multiple outcome measures, to reflect the multidimensional nature of pain, in order that clinically meaningful change in pain and inter-related outcomes are determined [19]. In keeping with this recommendation, secondary measures of symptoms of depression [Beck Depression Inventory (BDI)], health-related quality of life (HRQoL) [EuroQol 5-Dimensional outcome questionnaire (EQ-5D)] and self-efficacy [Self-efficacy for Managing Chronic Disease 6-Item Scale (SE-6)], were completed at Week 0 and each follow-up interview [26–28].

The BDI [26] measures symptoms of depression and is able to detect change over time [29, 30]. An isiXhosa version of the BDI-II has been found to be valid and reliable [31]. It has subsequently been used amongst rural South African amaXhosa people in the Eastern Cape and urban amaXhosa WLWHA [7, 32]. The BDI consists of 21 questions, each with four possible responses corresponding with a score (0–3), the sum of which is the total BDI score [26, 33]. The highest total score on the BDI is 63, with a higher score indicating greater depressive symptoms [26, 33].

The EQ-5D [27] generates two scores, one of which is the EQ-5D VAS score [27]. The EQ-5D VAS score measures an individual’s current health state using a scale of 0–100 where ‘0’ is the ‘worst imaginable health state’ and ‘100’ is the ‘best imaginable health state’ [27]. The isiXhosa version, the EQ-5D–Xhosa, is reliable and validated [34] and has been used in South African urban areas amongst amaXhosa PLWHA [7, 35] and in rural areas amongst people with people living with disability [7, 35, 36].

In self-management programmes, such as the PL programme for PLWHA, the SE-6 has commonly been used as a measure for change in self-efficacy [7, 28, 37]. The total SE-6 score, is determined by the mean of the six questions, which are each answered on a scale of one ‘not at all confident’ to ten ‘totally confident’ [28]. The SE-6-Xhosa is validated and has single factor structure with good internal consistency [38].

A post study structured interview was conducted telephonically after week 48 by the research interpreter. The series of open-ended questions differed for the two intervention groups for the purpose of discerning whether any contamination had taken place between the two groups and to better understand the impact of the interventions on each group.

### Recruitment and method

The recruitment and data collection process was piloted with five participants to establish its feasibility and acceptance for rural amaXhosa WLWHA. For the most part the pilot was satisfactory, although a problem regarding the BPI arose, where occasionally participants scored the pain severity ‘on average’ as higher than ‘at its worst’. To address this, additional training was done with the RA to develop standard explanations of the term ‘average’.

Following the pilot, recruitment commenced at the HIV outreach clinics at Pumalanga Clinic and Ngcwanguba CHC in February and March 2015. Women LWHA who indicated an interest in participating in the study, and who had completed pre- and post-HIV test counselling, were directed by medical officers, nursing staff and word-of-mouth at the clinic to a consulting room for the recruitment process. The principle investigator, together with the interpreter, screened for inclusion and exclusion criteria, including the ACSM health and fitness screening for exercise safety [17, 18]. Eligible women were invited to participate in the study, followed by the informed written consent process. All participants consented to participate in the study. If consent was given, demographic information was collected from participants, reviewing medical files where necessary, and a health literacy screening was completed to confirm that participants would be able to read the PL workbook. On completion of this recruitment process the baseline outcome measures were completed by the RA. The interpreter assisted the PI with all communication in isiXhosa with participants throughout the study.

To prevent contamination, participants from Pumalanga clinic were allocated to the PL intervention group, while participants from Ngcwanguba CHC were allocated to the TR intervention group. As the two clinics are located far apart geographically, interaction between the two communities is limited [39]. Participants were explicitly told to keep the intervention group they participated in unknown to the RA, who remained blinded throughout the study. The PL programme took place from Week 1 to Week 6, whilst the therapeutic relationship was developed during the data collection interactions which took place at baseline and each subsequent follow up visit with the RA.

Follow-up measures were obtained at Weeks 4, 8, 12, 24 and 48. For participants who were unable to attend the original follow-up date at each data collection point, a second date within 7 days was made available. If participants were still unable to attend, the option of doing a telephonic-based interview was given. Participants were informed that all transport costs would be reimbursed to cover the extra expense incurred due to study involvement and that during clinic visits for data collection points and PL programme sessions a small snack and a drink would be provided.

### Statistical analysis

The primary outcome as measured by the PSS and PIS of the BPI, showed that data were normally distributed using the Kolmogorov-Smirnov test (d = 0.08; *p* > 0.2) and therefore, parametric statistical analyses were conducted.

Distribution of all other data sets at baseline were also tested using the Kolmogorov-Smirnov test. Data for age, CD4 T-cell count at diagnosis and recent CD4 T-cell count, symptoms of depression, EQ-5D VAS and self-efficacy were all normally distributed. A pragmatic approach to statistical analysis was followed, where the distribution of the primary outcome measure is used to guide the analysis across all data [40]. In keeping with this pragmatic approach, parametric testing was conducted for the CD4 T-cell count, despite being commonly regarded as a non-parametric variable [40]. Accordingly, results are presented as means (M) and standard deviations (SD) throughout. Descriptive measures such as frequency tables, proportions and Pearson Chi-squared tests (χ2) were used to analyse categorical data.

Cumulative-link mixed model ordinal regression was used to test the effects of group, time, and group and time interactions on pain severity and pain interference. Linear mixed model regression was used to test the effects of group, time, and group and time interactions on depression and self-efficacy. A general additive mixed model with inflated beta distribution was used for HRQoL because the data were not normally distributed. These approaches were chosen over two-way analysis of variance (ANOVA) for significance testing because they are more robust when there are missing data. In all models the random effect was study participants. Diagnostic tests were performed on the linear mixed models to determine whether the data were appropriate for the use of linear regression modelling. If residuals were not normally distributed, data were transformed as necessary. All models were compared to each other and a null model (intercept only) using likelihood ratio tests. Four models, which were group, time, group and time, and group and time with a random slope, were assessed. If significance in a model was found, family-wise *p*-value correction was performed (Holm method) to reduce inflation of significance. If multiple models remained significant after correction, comparisons were made between these models using likelihood ratio tests to determine the best model fit. Thereafter, family-wise *p*-value corrections were repeated. If no significant model was found to be better, the simplest model was chosen.

Significance was accepted at *p* < 0.05 and analysis was by intention to treat. For mixed model regression and all other analyses, the original data set with missing data were used. R Version 3.1.2 was used for model regression analysis and included use of the ‘ordinal’, ‘lme4’, ‘GAMLSS’ and ‘car’ packages [41–45]. All other data were analysed by STATISTICA® [46].

## Results

**Fig. 1:**
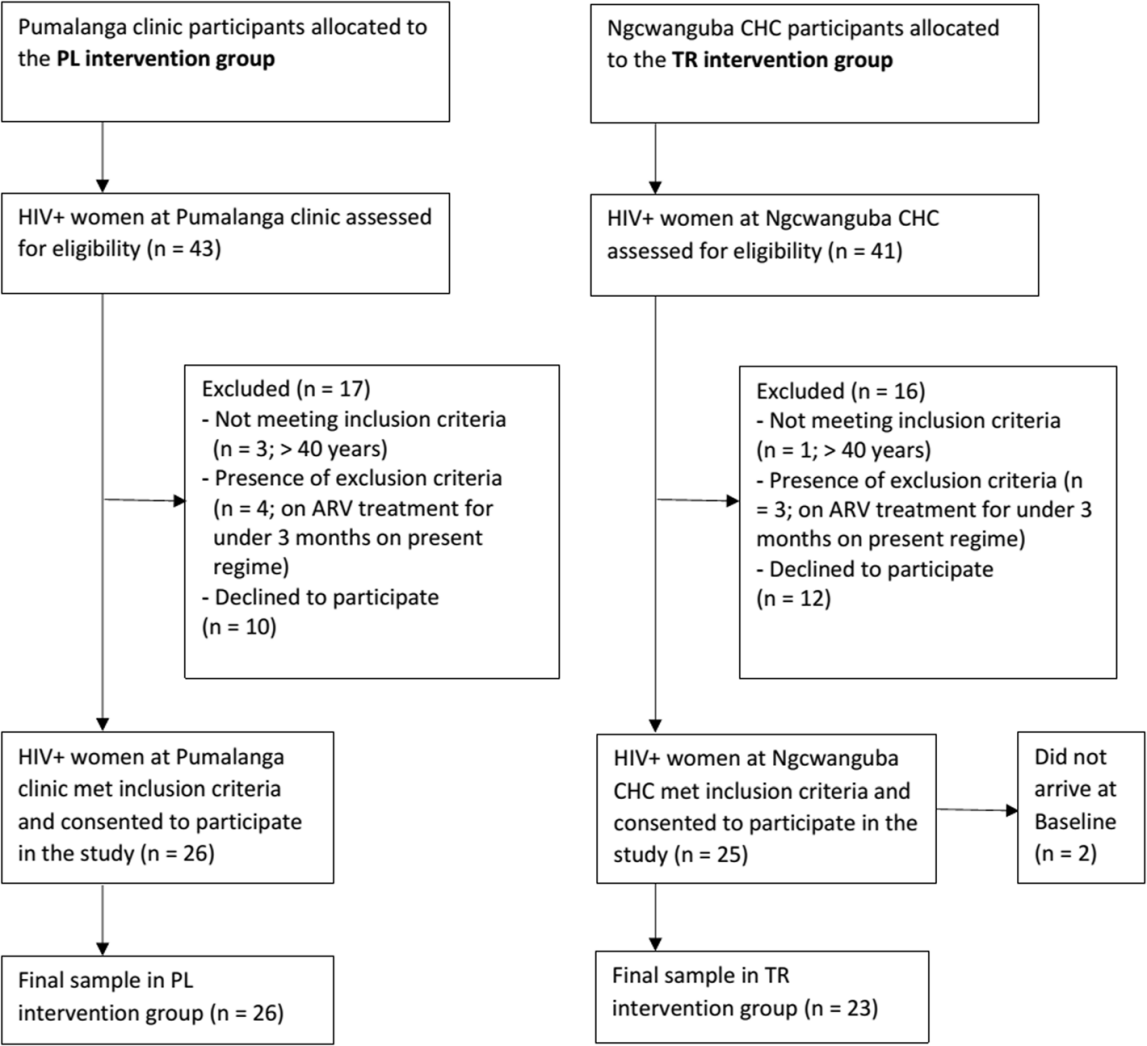
Screening, recruitment and allocation procedure

Twenty-six participants were recruited for the PL intervention group, which was split into two PL programme groups of 12 and 14. For the TR intervention group, 25 participants were recruited, of whom two participants did not arrive for baseline tests, leaving 23 participants in the final sample for the TR intervention group (Fig. 1).

### Attendance

**Fig. 2:**
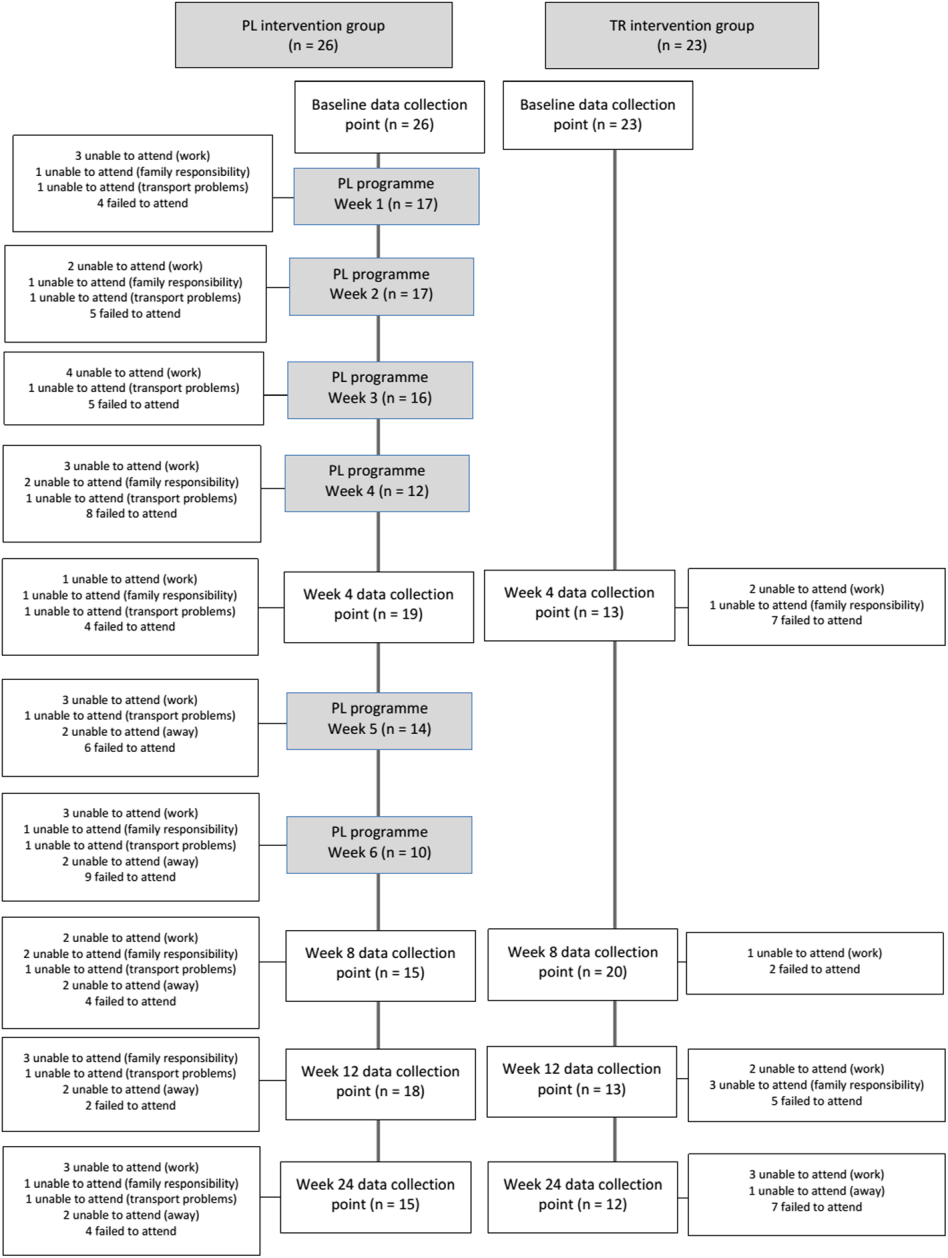
Attendance at PL programme and data collection points

Attendance fluctuated for various reasons (Fig. 2) for both the PL intervention group sessions (38–65% attendance of participants), and the follow up data collection points (Attendance PL group: 58–73%; Attendance TR group: 52–87%). Approximately half of the participants in the PL group (53%, 14/26) attended four or more of the PL group sessions.

Table 1 shows the socio-demographic and clinical characteristics of the groups. No significant differences were found between groups for age, employment status, highest level of education and health literacy. The entire study cohort had 8 to 9 years of education on average and over 80% were unemployed. All participants had limited health literacy, indicated by low self-reported reading ability, needing help frequently when reading health materials and a low level of education, all of which may have reduced the participants’ use of the PL workbook. Despite the significant difference between groups for CD4 T-cell count at diagnosis, no significant difference was found for the recent CD4 T-cell count, indicative of similarities between the groups in immune function at the time of the study.

**Table 1:** Socio-demographic and clinical characteristics of the PL and TR intervention groups at Week 0

	PL intervention group ***n*** = 26	TR intervention group ***n*** = 23	Significance Test
Age α	33 years ±5	32 years ±4	t = 0.72; *p* = 0.48
Employment β **Number (%)**			χ^2^ = 1.42; *p* = 0.7
**Unemployed not looking for work**	12 (46.2)	11 (47.8)	
**Unemployed looking for work**	9 (34.6)	8 (34.8)	
**Employed**	5 (19.2)	3 (13.0)	
**Unable to work –disability grant**	0 (0)	1 (4.3)	
Highest level of education β **Number (%)**			χ^2^ = 2.11; *p* = 0.55
**No schooling**	1 (3.8)	3 (13.0)	
**Primary education**	7 (26.9)	8 (34.8)	
**First 2 years of secondary education**	10 (38.5)	7 (30.4)	
**Last 3 years of secondary education**	8 (30.8)	5 (21.7)	
Years since diagnosis α			t = 0.81; *p* = 0.42
	4.3 ± 2.6	3.6 ± 3.9	
CD4 T-cell count at diagnosis α			t = 2.13; *p* = 0.04^a^
**Cells/μl**	206 ± 73	269 ± 112	
Recent CD4 T-cell count α			t = 0.52; *p* = 0.61
**Cells/μl**	478 ± 251	441 ± 227	
ARV management β **Number (%)**	n = 26	n = 23	χ^2^ = 0.42; *p* = 0.81
**Monitoring**	2 (7.7)	2 (8.7)	
**First-line**	22 (84.6)	18 (78.3)	
**Second-line**	2 (7.7)	3 (13.0)	

Note: number of participants (participants**,** PL intervention group, TR intervention group) varied for years since diagnosis (*N* = 47, *n* = 25, *n* = 22), CD4 T-cell count at diagnosis (*N* = 39, *n* = 23, *n* = 16) and recent CD4 T-cell count (*N* = 45, *n* = 25, *n* = 20)

α = mean ± SD, β = number (%)

^a^ indicates a significant difference between groups

### Pain severity score

**Fig. 3:**
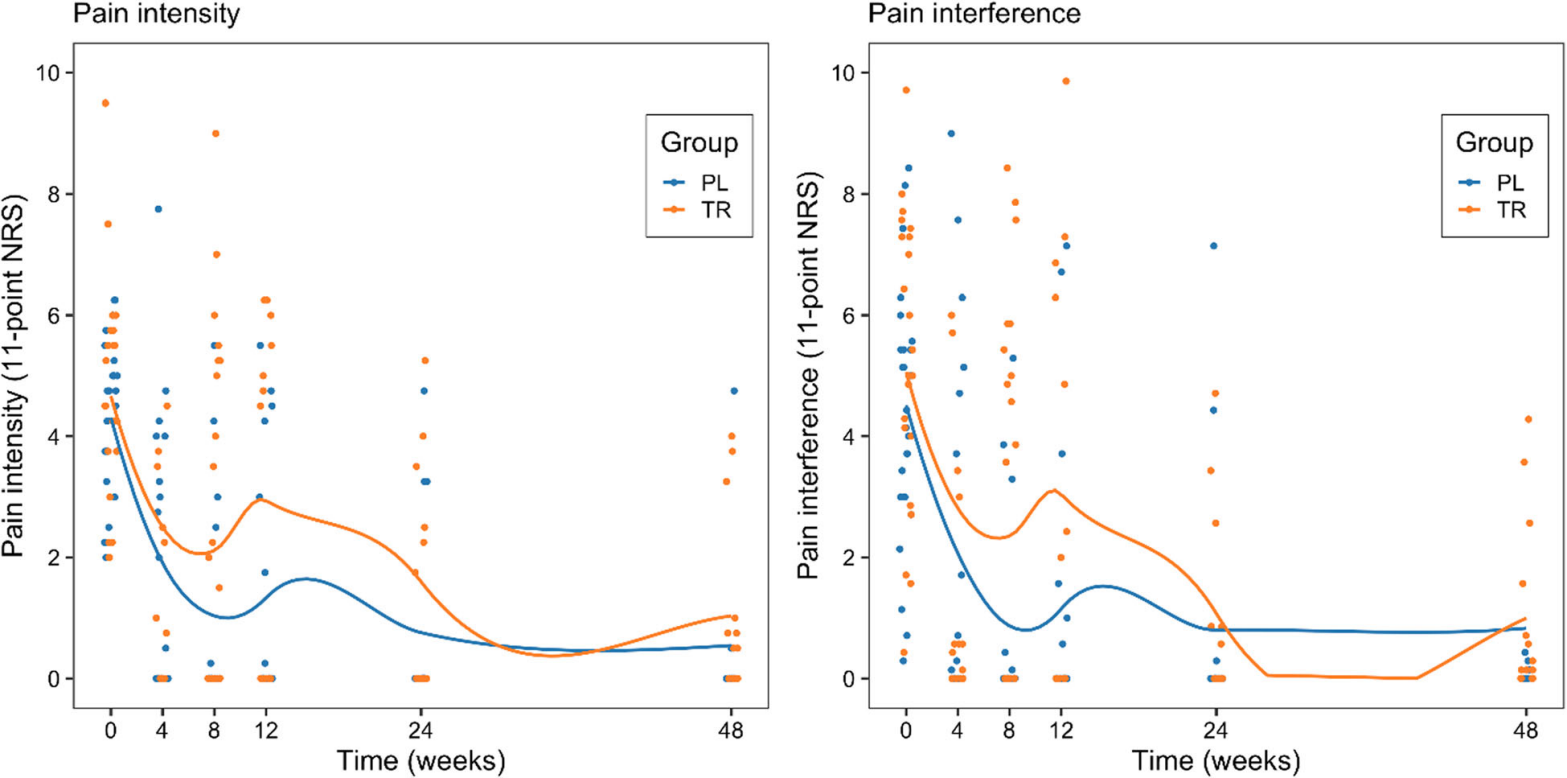
Change in Pain Severity Scores and Pain Interference Scores for both intervention groups over time Panel A: Pain Severity Scores: Change in Pain Severity Scores for PL and TR intervention groups over 48 weeks, (*N* = 49, PL intervention group (*n* = 26) and TR intervention group (*n* = 23)); Panel B: Pain Interference Scores: Change in Pain Interference Scores for PL and TR intervention groups over 48 weeks, *data missing for 1 participant for PIS scores, (*N* = 48, PL intervention group (*n* = 26) and TR intervention group (*n* = 23))

The mean PSS at Week 0 (baseline) for the sample was 4.57 (± 1.59), indicative of moderate pain, and no significant difference between groups existed (Table 2; t = 1.22; *p* = 0.23). A cumulative link mixed-model ordinal regression (Supplementary Table III) showed that the PSS in both groups reduced significantly over time (*p* < 0.001) and that a significant difference between the two interventions existed during the study period (*p* < 0.01), with the PL intervention group performing better. On inspection of the graph (Fig. 3, panel A), however, it appears that this difference occurred in the first eight weeks of the study after which the groups improved at a similar rate as determined by the mean lines being close together. With the mean PSS for the sample reducing by 3.31 points over the 48 weeks of the study, there was a clinically important reduction in pain severity for the sample over the study, regarded as a reduction of three points on a scale of 0–10 [19, 20, 47].

**Table 2:** Primary and secondary outcome scores for participants in PL and TR groups at Week 0

	PL intervention group *n* = 26	TR intervention group *n* = 23	Significance Test
PSS	4.3 (1.3)	4.9 (1.9)	t = 1.22; *p* = 0.23
PIS^a^	4.5 (2.2)	5.3 (2.4)	t = 1.19; *p* = 0.24
BDI	23.8 (10)	27.9 (12.2)	t = 1.3; p = 0.2
SE-6	6.4 (2)	5.8 (2.6)	t = 0.94; p = 0.35
EQ-5D VAS	66.7 (15)	52.2 (15)	t = 3.38; p < 0.01^a^

All values are mean (SD). ^a^data missing for 1 participant for PIS scores, (N = 48, n = 26, *n* = 22)

### Pain interference score

The mean PIS score at Week 0 for the sample was 4.87 (± 2.28), indicative of moderate interference. There was no significant difference between groups at Week 0 (Table 2; t = 1.19; *p* = 0.24). A cumulative linked mixed-model ordinal regression (Supplementary Table IV) showed that time was an independent predictor of PIS over the 48 weeks of the study (*p* < 0.001), with a significant reduction in PIS occurring in both the PL intervention group and the TR intervention group. Over time a significant difference between groups was also found, with the PL intervention group showing a greater decrease in pain interference. Similar to the PSS, the between group difference appears to have occurred in the first 8 weeks, however, after which the mean lines remain close together (Fig. 3, panel B). As the PIS of the sample reduced by 3.58 points over the 48 weeks of the study, these results can be considered as clinically important reductions in pain interference [19].

### Secondary outcomes

**Fig. 4:**
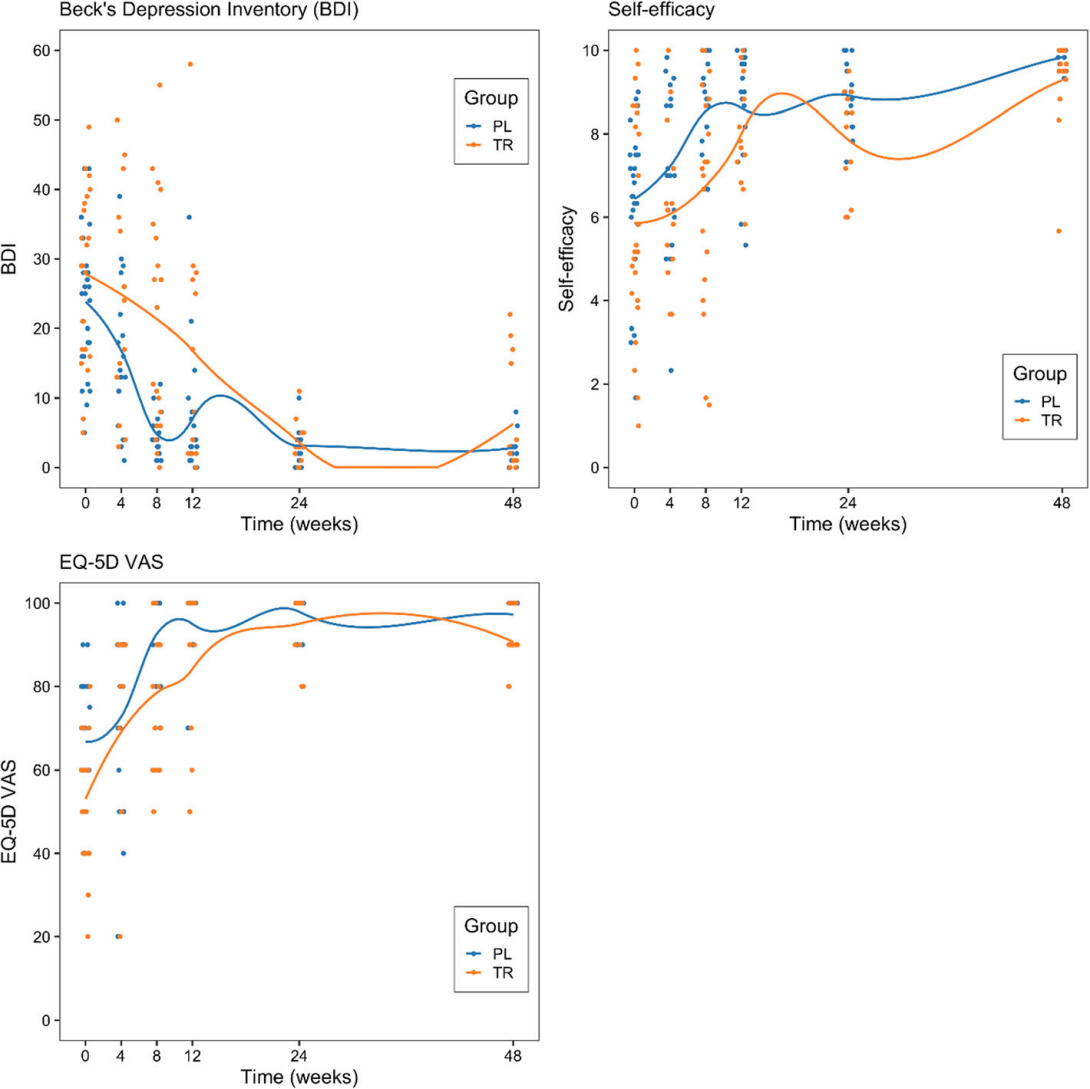
Change over time in scores on the Beck Depression Inventory, self-efficacy and EQ-5D VAS scores. Each dot represents an individual participant’s score; Panel A: Beck Depression Inventory: Changes in scores on the Beck Depression Inventory over time (*N* = 49); Panel B: Self-efficacy scores: Change in self-efficacy scores over time (*N* = 49); Panel C: EQ-5D VAS scores: Change in EQ-5D VAS scores over time (*N* = 49)

There were no significantly different baseline scores for BDI (Table 2; t = 1.3; *p* = 0.2) and self-efficacy groups (Table 2; t = 0.94; *p* = 0.35). The mean baseline score for the participants on the BDI was 25.73 (± 11.2), indicative of moderate depression. A linear mixed-model regression (Supplementary Table V) indicated an effect of time and group on the BDI score, where BDI and time were significantly inversely related (*p* < 0.001). A significant difference between groups existed with the PL intervention group having greater improvement (*p* < 0.01). The BDI score improved in both groups (Fig. 4, panel A), and on inspection of the graph it shows that after the first eight weeks both groups had similar scores, as with the change in the PSS and PIS over time.

For self-efficacy, the mean baseline score for the sample 6.15 (± 2.28). Self-efficacy scores improved over time (linear mixed-model regression; *p* < 0.001, and Supplementary Table VI) but there was no difference between the groups (Fig. 4, panel B).

A significant difference existed between groups at baseline for the HRQoL, where the PL intervention group scored significantly higher than the TR intervention group, as measured by the EQ-5D VAS (Table 2; t = 3.38; *p* < 0.01). From regression analysis (Supplementary Table VII) it was determined that HRQoL (Fig. 4, panel C) improved in all participants over the course of the study but there was no difference between the groups (EQ-5D-VAS, general additive mixed-model; *p* < 0.001).

### Interviews

The responses to the open-ended questions posed to participants at the end of the study were analysed for recurring topics. Participants from both intervention groups reported benefits in pain and symptom reduction, improved wellness and support gained. No hints of contamination between groups were found during analysis of the interview responses. Participants of the TR intervention group, despite not formally being a group, used the term ‘group’ to describe themselves in the post study interviews. This formation of a group, brought about by the participants themselves, was possibly as a result of attending follow up appointments at the same clinic and on the same day. Therapeutic relationship group participants referred to a positive environment in which they could share information and offer one another support and encouragement (Supplementary Table VIII and IX), which may have established a further care component.

## Discussion

This study was the first to assess the efficacy of a non-pharmacological intervention for pain in rural South African amaXhosa WLWHA. We investigated the efficacy of the PL programme combined with a therapeutic relationship, in comparison to a therapeutic relationship alone on pain severity and pain interference amongst rural amaXhosa WLWHA. We found that the PL and the TR intervention groups were both effective in significantly reducing pain severity, pain interference and depressive symptoms, and increasing self-efficacy and HRQoL over the 48 weeks of the study. Despite an apparent faster reduction in pain and depressive symptoms in the PL group within the first eight weeks (Figs. 3, 4), both groups had similar results after this point, which were maintained over the study period. What is noteworthy is that the significant reduction in symptoms was maintained over 48 weeks despite six months between the final two follow ups. Of further clinical relevance, was that over 50% of the participants in both groups had successful pain management, indicated by a clinically meaningful reduction in pain severity over the 48 weeks of the study. In contrast, there is no feasible pharmacological intervention which is more effective than placebo available in South Africa [5, 6]. The significant differences in reduction of symptoms in both groups, maintained over the 48 weeks of the study appear to be as a result of the therapeutic relationship, which was common to both groups. The implication of the results is that the implementation of the PL programme for rural amaXhosa WLWHA is not justified, given the similarity of results in both groups. However, effective pain management amongst rural amaXhosa women appears to be possible through the generation of a care factor or a therapeutic relationship.

In the first study of the Positive Living Programme [7], the care factor or therapeutic relationship was considered as a possible contributor to the significant reduction in pain severity and pain interference found in both the intervention group and those receiving only the PL programme workbook and follow-up [7]. In this study we purposefully generated the therapeutic relationship to determine its effect on pain and pain-related outcomes. All outcomes improved in the TR intervention group and were maintained for the study duration, emulating results of previous research indicating that health outcomes may be optimised by a therapeutic relationship [15]. Further, both groups in the current study showed significant improvement in pain severity and pain interference, emulating the results of Parker et al. [7]. This finding gives rise to further questioning of whether the results of Parker et al. [7], were a result of the therapeutic relationship or of the interventions of the PL programme and workbook. Similar results were found in a study comparing a self-management intervention to usual care in PLWHA in the United States of America (men and women) where no significant difference existed between groups for improvements in pain. In the self-management intervention group a reduction in pain severity of two points on the BPI was found, which may have been influenced by having peer and social support and a caring environment, which the participants reported receiving [48]. No mention of therapeutic relationships or social support were made by comparison in a study based in the United States of America (all male participants) comparing a self-management programme to usual care, where both groups show no significant improvements in pain and no significant difference was found between groups [49]. Being observed and cared for appears to contribute towards improvements in patient’s health outcomes and changes of behaviour [50], and these empathy-related changes may be mediated by the release of endogenous opioids and other neuroimmune interactions [15, 51–53]. The empathetic behaviour of the RA in the present study appears to have contributed to the overall effect of the reduction of pain in both groups of participants. Given the number of psychosocial factors associated with PLWHA and their experience of pain, that treatments for managing pain should be psychosocial in nature is supported [54].

The consistency of person as well as empathetic behaviour portrayed by the RA is a contrast to the fragmented care patients commonly experience in the South African public health care system and the impression of the clinic as a hostile environment [55]. The fragmented care, which arises due to high staff turnover and mobile patients [56] combined with the sometimes disrespectful health-professionals, results in dissatisfaction of patients with routine health care [55]. This study indicates that the provision of consistent empathetic care in the health care system, which assists the development of an ongoing therapeutic relationship, is beneficial and an effective pain management strategy for rural amaXhosa WLWHA.

A confounding factor arose in the therapeutic relationship group, however. This was the possible spontaneous formation of a group and the consequent provision of further social support. This phenomenon was not expected in these rural women and consequently was not measured. The formation of a group was also described in interview responses from the urban amaXhosa women in the control group of the previous study [7]. Some women reported forming their own informal groups, and receiving support from them. Thus, the extent of the therapeutic relationship alone on rural WLWHA is unclear. Notably, it appears that social support, whether from the purposeful development of a therapeutic relationship, or a group of peers, or a combination thereof, is effective for managing pain in rural amaXhosa WLWHA. Perhaps in these relatively healthy women, indicated by their clinical characteristics, who live with adversities such as high levels of unemployment, low levels of education and high likelihood of stigma attached to an HIV-positive status [57, 58], receiving care and support is most helpful for managing their pain.

The mechanism by which the clinical improvements came about is unclear due to multiple variables being inter-related [59, 60]. Increased self-efficacy has been associated with improvements in pain and depression in people living with painful conditions [61, 62]. Social support can facilitate a positive effect on health in people living with persistent or complex pain conditions by increasing self-efficacy and adaptive coping skills [63–66]. The development of a therapeutic relationship or the formation of a group provides an environment for social support, which may positively influence self-efficacy [22, 65]. As documented in literature and noted in the participants’ interview comments, social support within a group assists people living with chronic diseases with problem solving, establishing changes in health behaviour and leads to the development of self-efficacy [65, 67]. As people notice health improvements, this facilitates improvements in self-efficacy and leads to a greater sense of control, reduced fear and reduced symptoms [22, 65].

There were limitations to the study. The results of this study are not generalisable to all rural amaXhosa WLWHA but to women living in similar conditions to the areas around Zithulele and with similar health, as a result of the sampling method chosen. The study results may also not be applicable to amaXhosa men, urban amaXhosa women or to other cultural groups in South Africa or Africa. Attendance of the PL group sessions was low (38–65%) and so participants may not have received an optimal ‘dosage’. Despite such low attendance, including low attendance at the data collection points (attendance PL intervention group: 58–73%; attendance TR intervention group: 52–87%) benefits of both interventions were seen. These improvements despite suboptimal attendance are hopeful as they reflect the clinical scenario. That is, barriers to accessing health care, namely work, transport and family responsibility, appear to extend to the attendance of routine care generally in rural areas (C. Young, email communication, November 2016).

Considering these attendance barriers, fostering a care factor for rural amaXhosa WLWHA in a resource-poor environment may be more sustainable for significantly reducing pain compared to implementing an intervention such as the PL programme, with similar long-term results. Furthermore, the positive outcomes in this study were created with an RA and peer leader, both of whom were trained community members, and not qualified health care professionals. Subsequently, where health care professionals in this rural clinical setting have a high workload and are able to give less time to each individual to establish stronger therapeutic relationships, trained community members, such as the RA in this study would have more time devoted to this. In the South African health care system many forms of support staff exist to assist the health care available. Training community members for these roles, whilst maintaining clear communication that they are not trained health care professionals, may assist with the sustainability of such interventions in resource-poor environments and should be investigated further for its use in treatment.

## Conclusions

This research contributes to understanding the use of non-pharmacological interventions, particularly the effects of therapeutic relationships, to improve pain management for PLWHA, for whom pain is undermanaged internationally [1], and for vulnerable groups, such as women and people in poverty, who commonly receive poor pain management [2, 68–71]. The current research indicates that the purposeful development of a therapeutic relationship between a caring health care worker and a patient can bring about clinically meaningful changes in pain with significant reductions in pain severity and pain interference for amaXhosa WLWHA. The study results support the fostering of empathetic environments for rural amaXhosa WLWHA to reduce and manage pain. The development of therapeutic relationships and an empathetic environment is particularly germane in the South African health care system providing treatment for PLWHA to counter the impression of the clinic as a hostile environment [55], and to facilitate and allow for adequate pain management. The effect of the therapeutic relationship on both groups in this study are in keeping with other studies which indicate that the therapeutic relationship has a positive effect on both psychological and physical outcomes, including being able to adequately manage pain [15]. Future research should further consider the role of participation in a group and the role of social support for managing pain in rural amaXhosa WLWHA and other populations of PLWHA. Current recommendations are for health care professionals who work with rural amaXhosa WLWHA to place more emphasis on the care factor and develop skills to improve therapeutic relationships to facilitate improved pain management for their patients.

## Supplementary Information

**Additional file 1.**

## Data Availability

The datasets used and/or analysed during the current study are available from the corresponding author on reasonable request.

## Abbreviations

ACSM: American College of Sports Medicine
AIDS: Acquired Immune Deficiency Syndrome
ART: Anti-retroviral therapy
BDI: Beck Depression Inventory
BPI: Brief Pain Inventory
CBT: Cognitive Behavioural Therapy
CHC: Community Health Centre
EQ-5D: EuroQol 5-Dimensional outcome questionnaire
HIV: Human Immunodeficiency Virus
HRQoL: Health-related quality of life
IMMPACT: Initiative on Methods, Measurement, and Pain Assessment in Clinical Trials
NRS: Numeric rating scale
RA: Research assistant
PIS: Pain Interference Score
PL: Positive Living
PLWHA: People Living With HIV/AIDS
PSS: Pain Severity Score
SE-6: Self-Efficacy for Managing Chronic Diseases Six-Item Scale
TR: Therapeutic relationship
VAS: Visual Analogue Scale
WLWHA: Women Living With HIV/AIDS
