# Supplementary material for: Managing pain in HIV/AIDS: a therapeutic relationship is as effective as an exercise and education intervention for rural amaXhosa women in South Africa
Source: BMC Public Health. 2021 Feb 5;21:302. doi: 10.1186/s12889-021-10309-7 (PMC7866667; doi:10.1186/s12889-021-10309-7)
Supplement: Supplementary file 1 — Additional file 1. [file 12889_2021_10309_MOESM1_ESM.docx]

# Supplementary material

**Training method for peer-leader**

Training covered how to introduce the programme and each session, facilitate discussion on workbook educational topics, lead exercise routines and recognise adverse symptoms of exercise, facilitate relaxation techniques, and facilitate action planning and its modification. During training, the prospective peer-leaders acquired the skill of being able to demonstrate group facilitation through an experiential learning approach, and became familiar with using the workbook contents as a guide. Additionally, debriefing sessions were held with the peer-leader after each session during the PL programme intervention by the primary investigator. These served to ensure content veracity and skill maintenance, by the provision of further recommendations for the following sessions. Only one trained peer-leader was able to commit to the time frame of the PL programme and became the facilitator for the PL groups.

**Timeline for PL and TR intervention groups**

Figure 5: Procedure and timeline for the PL and TR intervention groups

**Supplementary tables**

**Supplementary Table III. Final model for Pain severity score**

|  | Estimate | Standard error | P value |
| --- | --- | --- | --- |
| TR group* | 0.85 | 0.32 | 0.008 |
| Time | -0.05 | 0.01 | <0.001 |

*Therapeutic relationship (TR) group relative to Positive Living (PL) intervention group

**Supplementary Table IV. Final model for Pain interference score**

|  | Estimate | Standard error | P value |
| --- | --- | --- | --- |
| TR group* | 0.82 | 0.35 | 0.02 |
| Time | -0.05 | 0.01 | <0.001 |

*Therapeutic relationship (TR) group relative to Positive Living (PL) intervention group

**Supplementary Table V. Final model for Beck’s Depression inventory**

|  | Estimate | Standard error | P value |
| --- | --- | --- | --- |
| TR group* | 0.85 | 0.31 | 0.007 |
| Time | -0.07 | 0.006 | <0.001 |

*Therapeutic relationship (TR) group relative to Positive Living (PL) intervention group

**Supplementary Table VI. Final model for Self-efficacy 6 scale**

|  | Estimate | Standard error | P value |
| --- | --- | --- | --- |
| Time | 0.95 | 0.09 | <0.001 |

**Supplementary Table VII. Final model for Euroqol-5D VAS scale**

|  | Estimate | Standard error | P value |
| --- | --- | --- | --- |
| Time | 0.03 | 0.005 | <0.001 |

**Interview responses**

**Supplementary Table VIII: Positive Living intervention group responses (n = 12)**

| **Topic** | **Question: Did you find the group useful? Please explain.** |
| --- | --- |
| **Pain alleviation** | Participant 2: *“Yes, I had a backache and it got better.”*  Participant 3: *“And when you are doing the exercises you don’t feel any pain.” AND “…I feel relieved…”*  Participant 9: *“Yes, because since I have been going to the group I feel alright, I don’t feel any pain.”*  Participant 26: “…*because there was a pain that I used to feel on my legs but I can’t feel it anymore.”* |
| **Exercise** | Participant 2: “*[What helped the pain] was doing the exercises.”*  Participant 3: *“Yes, a lot, because we were always doing the exercises. And when you are doing the exercises you don’t feel any pain. And when I wake in the morning I do the exercises and I feel relieved.”*  Participant 6: *“It is interesting because of the exercising we were doing…”*  Participant 26: *“Yes, …because of the exercises.”*  Participant 30: *“Yes, because I was lazy to do the things like going to the river, when I got the exercises I found it easy to go to the river and my life changed.”*  Participant 34: *“A lot … if I have a headache I must just take a bucket and go to the river. Even if I have pain, I just do the exercises then after I feel better.”* |
| **Improving wellness** | Participant 15: *“A lot. I felt alright because of the things she said we must do.”*  Participant 36: *“Yes. Because it made me to stop regretting myself. I felt alright and strong. It made me accept my situation.”*  Participant 42: *“Yes, it was helpful because when I don’t feel well I does the things that she said we must do.”*  Participant 46: *“Yes, it was helpful a lot. Because when you are doing the exercises you don’t feel any stress, you feel happy all the time you always healthy.”* |

| **Topic** | **Question: Did you share or discuss any of the information with anybody else? Please explain.** |
| --- | --- |
| **Sharing and support** | Participant 3: *“…I go to the support group…” AND “…she’s also HIV positive so I used to share the book with her and explain to her what’s the book about.”*  Participant 5: *“I discuss with the other people” AND “…I also make an example about myself.”*  Participant 15: *“…I explain to them about the things that I read in the book.”*  Participant 26: *“…I used to discuss with my group mates.”*  Participant 30: *“There is one person I discuss with…”*  Participant 34: *“I discuss a lot…”* |

**Supplementary Table IX: Participants’ responses from the TR intervention group (n = 12)**

| **Topic** | **Questions: Did you receive any information during the study about HIV/AIDS that helped you to manage your symptoms? Please explain.** |
| --- | --- |
| **Symptom alleviation** | Participant 48: *“…I noticed that my legs were very weak but now they are strong…” AND “…because others were very worse and were very weak but I noticed that they are active now, that group is very helpful.”* |
| **Exercise** | Participant 24: *“… [The group] even taught us how to do exercises…”* |
| **Sharing and support** | Participant 48: *“And being in the group with other people, sharing information you get more experience and I feel happy when I’m in the group with other people because we laugh and chat.”* |
| **Hopefulness and encouragement** | Participant 13: *“…I was always sad but I felt happy because I was encouraged and they told me that it’s not the end of life…” AND “I would love to encourage other people…”*  Participant 16: *“…it’s not the end of life and you can live for more than 30 years. It doesn’t mean you are unique from other people you are not different but you must take care of yourself.”*  Participant 23: *“…the questions [the RA] asked us like do we regret ourselves, questions like that are the ones that made me feel strong.”*  Participant 25: *“… [the RA] makes it easy for us to manage everything like telling us that we must not undermine ourselves.”*  Participant 35: *“… [the RA] tell us that we must not feel different from other people.”*  Participant 38: *“…I was really encouraged. And I don’t have any complaints because everything is going well.”* |

| **Topic** | **Questions: Did you discuss ways to manage symptoms with others? Please explain.** |
| --- | --- |
| **Support and acceptance** | Participant 13: *“If next year the group is still continuing I would love to encourage other people, I would tell people that this disease is not different from the other diseases and it’s not the end of life.”*  Participant 48: “*Yes with my [family]. I tell them that this is not the end of life and if they take care of themselves they will not be different from other people; they will live for a long time…”*  Participant 29: *“If someone is not alright I discuss with them like my sister–in-law…she used to hide her tablets and…I advised her and now she’s alright…”*  Participant 35: *“The only person I discuss with is my father. He couldn’t accept this disease; he is also HIV positive.”*  Participant 24: *“We discussed in the group about how to take care of ourselves.”*  Participant 23: *“Yes, …I discuss with [my friend] because she didn’t want to accept she is HIV positive so I advised her and now she’s alright.”*  Participant 38: *“Yes, with my sisters.”* |
